# Endocarditis caused by Scopulariopsis brevicaulis with disseminated emboli and multiple vascular aneurysms: A case report and literature review

**DOI:** 10.1016/j.idcr.2025.e02366

**Published:** 2025-09-13

**Authors:** Kieffer Korvin, Myriam Chiarruzi, Aurélie Martin, Clara Gromoff, Vlad Ciobotaru, Milène Sasso, Marie-Clothilde Brunet, Romaric Larcher, Paul Loubet

**Affiliations:** a Department of Infectious and Tropical Disease, CHU Nîmes, Nîmes, France; b Department of Cardiology, CHU Nîmes, Nîmes, France; c Department of Microbiology, CHU Nîmes, Nîmes, France; d Department of Cardiac Surgery, CHU Montpellier, Montpellier, France; e Virulence Bactérienne et Infections Chroniques, INSERM U1047, Univ Montpellier Department of Infectious and Tropical Disease, CHU Nîmes, Nîmes, France

**Keywords:** Scopulariopsis brevicaulis, Endocarditis, Olorofim, Antifungals

## Abstract

**Background:**

*Scopulariopsis brevicaulis*, a saprophytic fungus often associated with onychomycosis, has seldom been reported to cause deep-tissue infections in immunocompromised patients.

**Objectives:**

To review the antifungal susceptibility of *S. brevicaulis* and management strategies for related endocarditis and other invasive infections.

**Sources:**

A literature search was conducted using PubMed until September 2024.

**Content:**

This review presents a case of *S. brevicaulis* prosthetic valve endocarditis, emphasizing the diagnostic challenges and clinical implications of disseminated infection.

A summary of published cases of endocarditis and other deep-tissue infections is included along with a discussion on the antifungal susceptibility of *S. brevicaulis* and current treatment strategies.

**Implications:**

*S. brevicaulis* presents a significant treatment challenge due to its rare and opportunistic nature, along with frequent multi-drug resistance. Although there is no single optimal therapy for this mold, a practical approach combines medical and surgical strategies customized to individual cases, employing a variety of agents based on in vitro sensitivity testing. Determining minimum inhibitory concentrations (MICs) is essential for guiding treatment decisions, even in the absence of established clinical breakpoints for this pathogen. Olorofilm, a novel oral antifungal, demonstrates remarkable potential for treating *S. brevicaulis.*

## Introduction

*Scopulariopsis brevicaulis*, a saprophytic fungus commonly linked to onychomycosis, has rarely been documented as a cause of deep-tissue infections in immunocompromised patients. Its role in fungal endocarditis is rare, making this case particularly unique and worthy of attention. The review highlights the difficulties in diagnosing and treating *Scopulariopsis brevicaulis* due to the absence of established guidelines.

## Case report

The patient was a 61-year-old man with a medical history of bicuspid aortic valve, hypertension, hypercholesterolemia, gout, and sleep apnea. In 2020, he developed chronic kidney disease secondary to an unclassified vasculitis, with a baseline glomerular filtration rate (GFR) of 33 mL/min. Extensive investigations failed to identify the etiology of the vasculitis, and he had never received immunosuppressive therapy.

In July 2022, the patient had a programmed aortic valve prosthetic implant (bicuspid aortic valve) and supra coronary aortic replacement (for an aortic aneurysm).

From November 2022 to March 2023 (4 months following his surgery), the patient developed progressively increasing symptoms, including fatigue, arthralgia (initially diagnosed as gout), myalgia, rhabdomyolysis, fever, as well as a biological inflammatory syndrome.

**Fig. 1:**
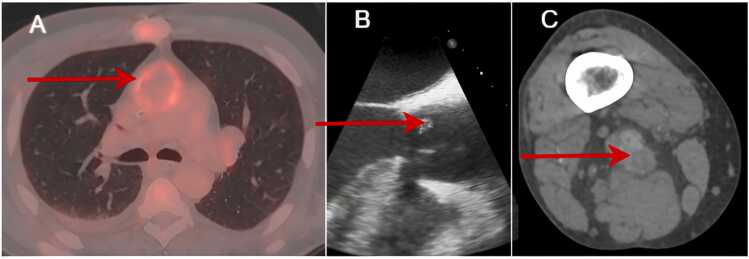
Medical imaging results.

In this context, the diagnosis of Endocarditis was suspected, but all blood cultures were negative, and multiple transthoracic and transesophageal echocardiogram (TEE) showed no sign. The fluorine-18-labeled fluorodeoxyglucose-positron emission tomography/ computed tomography (PET/CT) revealed hypermetabolism in the aortic valve and aortic tube, along with several mediastinal and splenic lymphadenopathies (Fig. 1**-A**).

All other microbiological investigations returned negative results, including serologies for *Coxiella*, *Bartonella*, *Brucella*, *Legionella* serologies, and *Tropheryma whipplei* blood and stool PCR. Anti-neutrophil cytoplasmic antibodies (ANCA) and antinuclear antibodies (ANA) also tested negative.

Initially considered bacterial endocarditis, the patient received multiple ineffective antibiotics, including Daptomycin, Dalbavancin, Meropenem, and Vancomycin combined with Doxycycline.

In November 2023, the patient experienced multiple vascular complications, including several strokes and septic emboli of the upper and lower right limbs. A new TEE showed an abscess around the aortic bioprosthetic, which also showed early degeneration resulting in valvular dysfunction.

In December 2023, a lymph node biopsy revealed a Hodgkin lymphoma, and the patient was admitted to the Hematology department of our University Hospital. However, the decision was made not to treat the lymphoma until the concurrent infection was resolved.

The patient was subsequently transferred to our Infectious Diseases ward. Beta-D-glucan was highly positive at 337 pg/mL (with a threshold of 7 pg/mL), indicating a fungal infection. Serum galactomannan and Aspergillus PCR tests were negative. Following these results, we initiated empiric antifungal therapy with intravenous liposomal Amphotericin B at 3 mg/kg/24 h. An abdominal CT scan identified a renal artery aneurysm on the verge of rupture, which was embolized as an emergency procedure.

A new PET/CT scan revealed a popliteal multilocular abscess in the lower right limb (Fig. 1-B&C) accessible for echo-guided puncture, leading to the microbiological diagnosis.

**Fig. 2:**
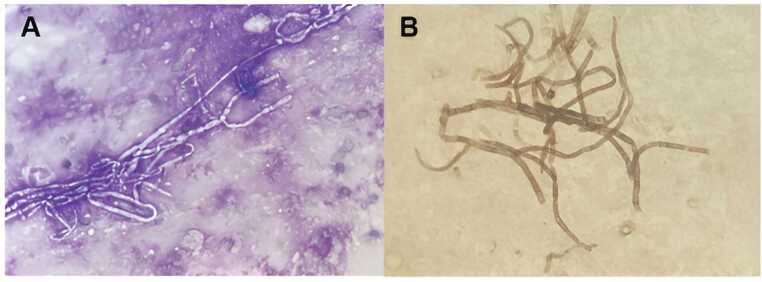
Microscopic aspects of the mold.

The direct examination revealed septate hyphal elements on direct microscopy examination after May- Grünwald-Giemsa staining procedure (Fig. 2.A) and Grocott’s methamine silver staining procedure (Fig. 2**.B**). The sequencing of the fungal internal transcribed spacer 2 (ITS2) region identified *Scopulariopsis brevicaulis* with 100 % similarity to the database (culture remained negative).

Following the guidance of the French National Reference Center for Invasive Fungal Infection, we increased the dosage of Liposomal Amphotericin B to 6 mg/kg/24 h and added Micafungin at 140 mg/24 h. This empirical regimen was initiated on expert advice and previous case reports, while antifungal susceptibility testing was still pending at the reference center.

**Fig. 3:**
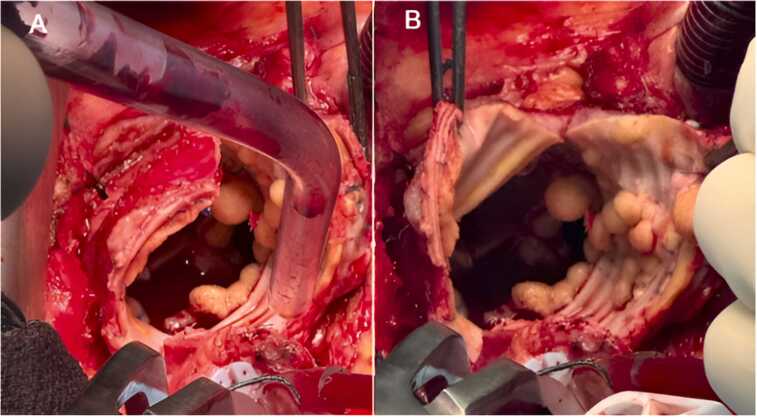
Macroscopic features of the supra coronary aortic tube during cardiac surgery.

The endocarditis team approved the surgical indication due to the high embolic risk and the significant risk of failure of medical treatment in this case. The patient underwent surgery in mid-January 2024, and the macroscopic appearance revealed a massive fungal proliferation inside the aortic tube prothetic (Fig. 3).

The postoperative course was characterized by cardiac tamponade, which was drained, and a brief cardiac arrest, from which the patient recovered sinus rhythm following cardiac massage. In the subsequent days, a hepatic artery mycotic aneurysm on the brink of rupture was discovered, which was embolized at the expense of sacrificing the superior hepatic artery, and there was an occlusion of the superior mesenteric artery responsible for digestive ischemia. The patient subsequently developed severe hepatocellular failure, which progressed to neurological deterioration with persistent unresponsiveness. He ultimately died at the end of January 2024.

Notably, the intraoperative samples tested culture-positive for *Scopulariopsis brevicaulis*. Although there are no clinical breakpoints for this pathogen, the MICs were determined via E-test at the Mycology laboratory and sent to the national reference center (Institut Pasteur, Paris) for broth microdilution according to the European Committee on Antimicrobial Susceptibility Testing (EUCAST) method. The E-test MIC for Isavuconazole was the lowest at 0.75, and all of the EUCAST MICs exceeded the limits of the tested antifungals (Table 1).

**Table 1:** Susceptibility testing of the S. brevicaulis isolate: E-test and European Committee on Antimicrobial Susceptibility Testing (EUCAST) MIC values.

	**E-test MICs (mg/L)**	**EUCAST MICs (mg/L)**
**Amphotericin B**	> 32	> 4
**Voriconazole**	> 32	> 8
**Posaconazole**	6	> 8
**Isavuconazole**	0.75	> 4
**Itraconazole**	-	> 8
**Caspofungine**	-	> 4
**Micafungine**	-	> 4

## Discussion

We reviewed the PubMed database without date restrictions until September 2024 for (i) data on the antifungal sensitivity of *Scopulariopsis brevicaulis* and (ii) all reports of invasive infections caused by *Scopulariopsis brevicaulis.*

### Antifungal sensitivity

There are few mycological studies on this species of fungi, and most focus on skin and tegument infections, primarily onychomycosis. Table 2 presents MIC ranges for some antifungals. In summary, no antifungal showed consistent susceptibility *in vitro*. Azoles appear to have very limited activity, while Amphotericin B and Terbinafine are more frequently susceptible.

**Table 2:** Susceptibility of *Scopulariopsis brevicaulis* to antifungals.

**Drug**	**MIC Range (µg/mL)**						
	*Skóra* et al[1](n = 40)	*Odero* et al[2]. (n = 10)	*Cuenca-Estrella* et al[3]. (n = 25)	*Yao* et al[4](n = 11)	*Aguilar* et al[5].(n = 5)	*Sandoval-* *Denis* et al[6]. (n = 48)	*Wiederhold* et al. (n = 59)
**Method**							
**Antifungal**	*CLSI*	*CLSI*	*CLSI*	*CLSI*	*CLSI*	*CLSI*	*CLSI*
**Fluconazole**		≥ 128			> 64		
**Itraconazole**	≥ 16	1 - ≥ 16	> 8 MIC_90_ > 8		> 16	1–32 MIC_90_ 32	
**Voriconazole**	8 - ≥ 16	1 - ≥ 8	> 8 MIC_90_ > 8			2–32 MIC_90_ 32	0.06 –> 16 MIC_90_> 16
**Posaconazole**		1–4	> 8 MIC_90_ > 8	> 16		1–32 CMI_90_ 32	0.5–> 16 MIC_90_> 16
**5-fluorocytosin**		≥ 64			> 128		
**Caspofungin**			> 16 MIC_90_ > 16	> 16		1–16 MIC_90_ 16	0.016–> 8 MIC_90_ 8
**Anidulafungin**						0.25–16 MIC_90_ 16	
**Micafungin**					4 - > 16	0.06–16 MIC_90_ 16	
**Terbinafine**	0.5–16		4 - > 16 MIC_90_ > 16	4 - > 16		0.5–4 MIC_90_ 2	
**Amphotericin B**	4 - > 16	1 - ≥ 8	8 - > 16 MIC_90_ > 16		1 - > 16	2–32 MIC_90_ 32	0.06–16 MIC_90_ 16
**Olorofilm**							≤ 0.008– 0.5 MIC_90_ 0.125

**CLSI:** Broth microdilution method performed according to CLSI document M38-A2. **MIC range:** the lowest and highest minimum inhibitory concentrations (MICs) observed among all tested isolates. **MIC₉₀:** the MIC value at which 90 % of the isolates are inhibited.

### Antifungal synergy

An *in vitro* study including 11 *S. brevicaulis* isolates demonstrated an 81 % synergistic interaction with the combination therapy of Amphotericin B and Anidulafungin and even greater activity when incorporating Terbinafine into a triple antifungal therapy [7].

Another study involving 25 strains demonstrated synergistic activity of combination therapies, particularly with Posaconazole and Terbinafine, and also for certain strains with amphotericin B plus caspofungin, posaconazole plus caspofungin, and voriconazole plus caspofungin [3].

### New antifungals

Olorofilm is a new antifungal agent with a novel mechanism of action that interferes with pyrimidine biosynthesis in fungi by inhibiting the dihydroorotate dehydrogenase (DHODH) enzyme. In a recent study, this agent showed good *in vitro* activity against *Scopulariopsis* isolates (n = 59), with a MIC range of ≤ 0.008–0.5 mg/L and MIC90 (MIC value at which 90 % of the isolates are inhibited) measured at 0.125 mg/L [8]. This drug may offer a promising option for this difficult-to-treat fungus.

### Treatment guidelines

The European Confederation of Medical Mycology's (ECMM) 2021 global guidelines recommend first-line treatment with Isavuconazole or Voriconazole, with L-AmB as a possible alternative—alone or in combination with Voriconazole. For salvage treatment, the guidelines suggest Posaconazole, which may also be used with micafungin alone or in combination with Terbinafine [9].

European Society of Clinical Microbiology and Infectious Diseases (ESCMID) and the European Confederation of Medical Mycology (ECMM)’s joint guidelines from 2014 recommend itraconazole and liposomal amphotericin B, although the evidence quality is low. Surgical treatment is also suggested, with any antifungal being the most preferred option therapy [10].

### Endocarditis and other invasive infections

#### Endocarditis

We identified seven cases of endocarditis in patients with prosthetic valves, who were mostly immunocompetent (**Supplementary Table 1**) [11], [12], [13], [14], [15], [16], [17]. The main characteristics included a slow onset of disease, large vegetations at the time of diagnosis, and common thrombotic, embolic, and vascular complications. Treatment approaches were both surgical and medical, with a diverse range of antifungals employed, including Voriconazole, Itraconazole, Posaconazole, Liposomal Amphotericin B, Caspofungin, and Micafungin.

A systematic review published in 2021 identified only three cases of *Scopulariopsis* endocarditis among 250 reported fungal endocarditis cases, highlighting the rarity of this entity [18].

#### Other invasive infections

The other recent reported invasive infections occurred in patients with severe cellular immunosuppression, except for one case of external necrotizing otitis in a young immunocompetent individual. The cases included fungemia, pneumonia (which presents similarly to Aspergillus), necrotizing skin lesions, endophthalmitis, and necrotizing sinusitis (**Supplementary Table 2**) [19], [20], [21], [22], [23], [24], [25], [26], [27], [28]. Medical treatment involves a combination of different antifungals. Two deaths occurred in patients undergoing stem cell transplantation due to pneumonia.

## Conclusion

Filamentous endocarditis is difficult to diagnose and should be considered in cases of culture-negative endocarditis among patients with prosthetic heart valves or endovascular devices, particularly in the presence of vegetation and/or multiple embolisms and aneurysms. In such complex cases, measuring beta-D-glucan levels can aid in guiding the diagnosis. *Scopulariopsis brevicaulis* is a rare opportunistic filamentous fungus that poses treatment challenges due to its frequent multi-drug resistance. There is no definitive optimal therapy for this mold, but it should typically be medico-surgical, involving a combination of various agents based on in vitro sensitivity. Determining MICs is important, even though there are no approved clinical breakpoints for this pathogen. Olorofilm, a new antifungal agent, shows promise for treating these fungi

## Ethical approval

not applicable

## Consent

The patient's relatives consented to the publication of this case report.

## Author contribution

KK (conception, data collection, writing, and review of manuscript), MC (review of manuscript), AM (review of manuscript), CG (review of manuscript), MS (writing and review of manuscript), RL (review of manuscript), PL (conception, supervision, writing, and review of manuscript).

## Funding

None

## Author statement

Our data are unavailable because they include confidential patient information.

## CRediT authorship contribution statement

**Paul Loubet:** Writing – review & editing, Supervision, Conceptualization. **Romaric Larcher:** Writing – review & editing. **Milène Sasso:** Writing – original draft. **Clara Gromoff:** Writing – review & editing. **Aurélie Martin:** Writing – review & editing. **Chiaruzzi Myriam:** Writing – review & editing. **Kieffer Korvin:** Writing – original draft, Data curation, Conceptualization.
